# Community action research track: Community-based participatory research and service-learning experiences for medical students

**DOI:** 10.1007/s40037-017-0397-2

**Published:** 2018-01-26

**Authors:** Nora Gimpel, Tiffany Kindratt, Alvin Dawson, Patti Pagels

**Affiliations:** 1 Community Health Section, Department of Family and Community Medicine, University of Texas Southwestern Medical School, Dallas, TX USA; 2 Department of Physician Assistant Studies, University of Texas Southwestern School of Health Professions, Dallas, TX USA

**Keywords:** Service-learning, Community-based participatory research, Family medicine, Medical school, Medical education, Postgraduate, Underserved, Curriculum

## Abstract

Community-based participatory research (CBPR) and service-learning are unique experiential approaches designed to train medical students how to provide individualized patient care from a population perspective. Medical schools in the US are required to provide support for service-learning and community projects. Despite this requirement, few medical schools offer structured service-learning. We developed the Community Action Research Track (CART) to integrate population medicine, health promotion/disease prevention and the social determinants of health into the medical school curriculum through CBPR and service-learning experiences. This article provides an overview of CART and reports the program impact based on students’ participation, preliminary evaluations and accomplishments. CART is an optional 4‑year service-learning experience for medical students interested in community health. The curriculum includes a coordinated longitudinal program of electives, community service-learning and lecture-based instruction. From 2009–2015, 146 CART students participated. Interests in public health (93%), community service (73%), primary care (73%), CBPR (60%) and community medicine (60%) were the top reasons for enrolment. Significant improvements in mean knowledge were found when measuring the principles of CBPR, levels of prevention, determining health literacy and patient communication strategies (all *p*’s < 0.05). Most students (73%) were satisfied with CART. Projects were disseminated by at least 65 posters and four oral presentations at local, national and international professional meetings. Six manuscripts were published in peer-reviewed journals. CART is an innovative curriculum for training future physicians to be community-responsive physicians. CART can be replicated by other medical schools interested in offering a longitudinal CBPR and service-learning track in an urban metropolitan setting.

## Introduction

Clinical prevention and population health training is a national priority for medical schools [1]. Curricula should comprise lecture-based and experiential training on: population medicine, health promotion/disease prevention and social determinants of health [2]. Community-based participatory research (CBPR) and service-learning are unique approaches to train medical students how to provide patient care from a population perspective.

CBPR includes community stakeholders in all phases of research [3]. Academic researchers partner with community organizations to determine how to best meet their needs by building on their strengths and integrating knowledge to meet shared goals. For example, leaders from a clinic providing care to uninsured patients collaborated with a student to evaluate its patients’ medical needs. Hypertension was identified as the most prevalent condition and no-show rates were high among those patients. A follow-up study aimed to determine their barriers to care.

Service-learning is a structured experience that combines community service with specific learning objectives, preparation and reflection through community-academic partnerships [4]. This process aims to address issues that communities face. Examples include providing vaccines for children at homeless shelters and conducting health fairs to link patients to medical homes.

The Liaison Committee on Medical Education has recognized the value of community-academic partnerships in its Standards for Accreditation of Medical Education Programs Leading to the MD Degree stating that medical schools must provide support for service-learning and community projects (Element 6.6) [5]. From 2014–2015, only 19 out of 126 (15%) programs in the US offered service-learning [6]. Furthermore, students indicated several areas of educational and experiential need regarding the intersection of health outcomes and social/structural factors that affect the health of their communities [7]. In 2016, only 31% of students reported participation in community-based projects [7].

We developed the Community Action Research Track (CART) as an innovative response for integrating population medicine and health promotion/disease prevention into the medical school curriculum through CBPR and service-learning. Goals of CART are to:

1. provide a comprehensive educational experience which incorporates existing curricular offerings, CBPR and service-learning and
2. increase the number of medical students with knowledge of health promotion/disease prevention, population medicine, CBPR, and social determinants of health.

Objectives of this article are to:

1. describe the CART curriculum;
2. evaluate changes in knowledge;
3. assess program satisfaction; and
4. describe students’ accomplishments.

## Methods

### Setting and participants

Established in 2009, CART is an optional 4‑year experience for medical students interested in community health at the University of Texas Southwestern (UTSW) Medical School. Students (~200 enrolled annually) participate in 2 years of lecture-based instruction followed by 2 years of clinical rotations. CART students are recruited upon enrolment. Program materials are mailed to students and presented at the new student organization fair. An orientation providing curriculum overview, requirements and examples of previous CART student projects is held after courses commence. Current CART students attend and answer questions. Students can apply during their first to third years and typically enter the program before their third year. Most CART students participated in service-learning during their undergraduate education and are enrolled in the combined MD/MPH degree program. CART is part of a pipeline of service-learning and CBPR training programs which extends from medical school into residency [8, 9].

## CART curriculum

**Fig. 1:**
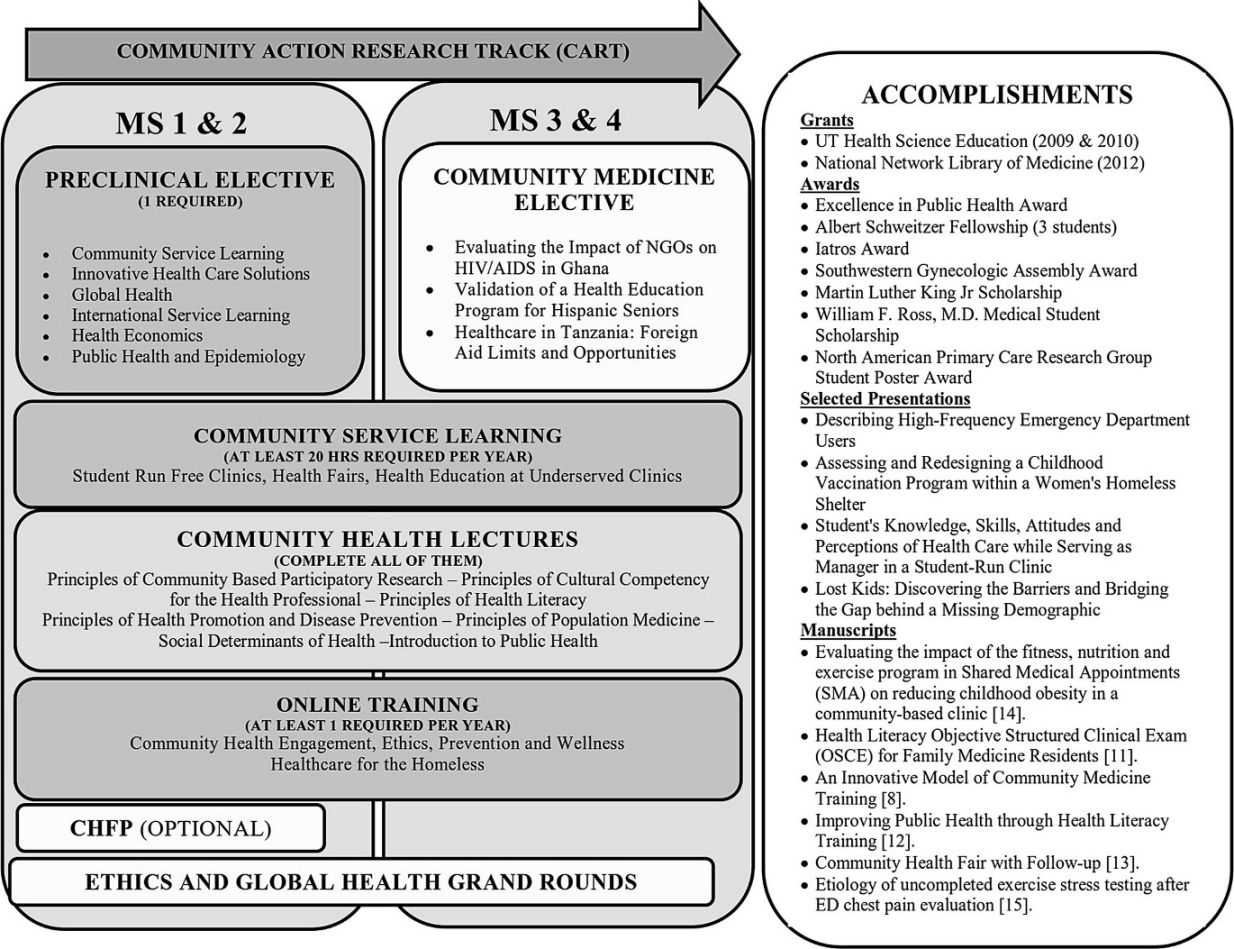
Overview of Community Action Research Track (CART) Curriculum (*CHFP* Communication Health Fellowship Program)

Components of CART and project examples are presented in Fig. 1. The curriculum comprises a coordinated longitudinal program of electives, service-learning and lecture-based instruction. Optional activities include the Community Health Fellowship Program, self-directed CBPR and grand rounds.

### Electives

CART students complete pre-clinical and community medicine electives. Pre-clinical electives (10–12 contact hours each) are completed during their first or second year. Course availability varies annually. The community medicine elective is a 4-week self-designed elective taken during their third or fourth year. Students participate in inter-professional experiences to improve the health status of community members in collaboration with local, national or international organizations. Many students complete CBPR projects during this elective.

### Community service-learning

CART students are required to complete 80 h of service-learning (20 h recommended annually). Students provide medical and non-medical services to underserved communities. Examples include volunteering at primary care clinics embedded in homeless shelters and providing nutrition and exercise education to primary school children to prevent childhood obesity.

### Lecture-based and online instruction

CART students are required to complete seven community health lectures and four online trainings. Lectures are developed and presented by inter-professional speakers and designed to introduce students to psychosocial, cultural and political dimensions of health. Online trainings promote reflective thoughts regarding the principles of CBPR and its intersection with the social determinants of health.

### Additional experiences

A subset of CART students participate in the Community Health Fellowship Program between their first and second year of medical school. This is a 9-week intensive CBPR training program where students gain advanced CBPR knowledge. A description of this program has been published elsewhere [10]. Students may participate in ethics and global health grand rounds.

## Knowledge, satisfaction and accomplishments

To determine changes in knowledge, brief surveys were administered before and after lectures (3–4 items). We developed survey measures based on learning objectives provided by each lecturer and items were measured using Likert scales (1 = strongly disagree; 5 = strongly agree). Students completed surveys on paper. Mean changes were calculated and Wilcoxon signed-rank tests conducted to determine statistically significant differences. To determine satisfaction, students completed an online survey which evaluated their motivations for enrolling into CART, their satisfaction with knowledge gained from CART and their satisfaction with the overall program. Evaluation items were measured using 5‑point Likert scales (1 = extremely dissatisfied; 5 = extremely satisfied). Responses were dichotomized (extremely satisfied/satisfied vs. other) and descriptive statistics were conducted. To describe student accomplishments, the total number of projects completed, grants awarded, projects presented and peer-reviewed publications were tracked.

## Results

From 2009–2015, we recruited 146 CART students. Most (80.1%) were female and 15% participated in the Community Health Fellowship Program. The proportion of students who completed CART requirements varied per graduating class. In 2010, 65% of students who graduated completed all requirements compared with 50% in 2012.

### Changes in knowledge

CART students’ self-reported knowledge improved on all items for cultural competency, public health, population health, CBPR, health promotion, health literacy and social determinants of health lectures. Significant improvements in mean knowledge were found when measuring strategies for taking a medical history, major branches of public health, mandatory reporting, the health care system safety net, principles of CBPR, levels of prevention, determining health literacy and patient communication strategies (all *p*’s < 0.05). No significant increases were found when evaluating the value of community-based projects in public health and the impact of social determinants of health outcomes.

### Satisfaction

Students (*n* = 35) reported many reasons for enrolling into CART. Interests in public health (93%), community service (73%), primary care (73%), CBPR (60%) and community medicine (60%) were the top five reasons for enrolment. Most students (73%) were extremely satisfied/satisfied with the program. Students were most satisfied with knowledge gained in community service-learning (95%) and health disparities (75%).

### Accomplishments

From 2009–2015, projects were disseminated by at least 65 posters and four oral presentations at local, national and international professional meetings. Three grants were funded and six manuscripts were published in peer-reviewed journals [8, 11–15]. Two CART students entered the UTSW Family Medicine Residency program to continue their CBPR and service-learning experience through our pipeline. Examples are presented in Fig. 1.

## Discussion

Our objectives were to describe the CART curriculum, evaluate changes in knowledge, assess program satisfaction and describe students’ accomplishments. Overall, we found that our curriculum improved students’ knowledge of community and population health concepts in several areas, students were satisfied with the program and students were successful at disseminating their results. Our results are similar to other studies evaluating medical student service-learning experiences [4].

CART is a replicable and nationally applicable experience for training future physicians to be aware of social, psychological, economic and social determinants of health by participating in comprehensive service-learning and CBPR experiences. CART provides students the opportunity to contribute to reducing and eliminating health disparities. The program links the medical school to the community and introduces students to the population health perspective through active community engagement. Several service-learning curricula exist in medical schools which involve both short-term and longitudinal training experiences [4]. Few programs offer training across the 4 year spectrum of medical school. To our knowledge, no programs offer a longitudinal CBPR and service-learning track in an urban metropolitan setting.

### Strengths and limitations

A strength of CART is the ability to provide students an opportunity to participate in service-learning and CBPR and disseminate their results. Almost half (47%) of the students presented results at professional meetings. Limitations of CART were insufficient resources and high turnover of support staff which resulted in a lack of regular evaluations and inconsistent tracking. Student satisfaction data was only available from 2012.

### Next steps

To address our limitations, we are developing a database to track activities and accomplishments. We are improving our evaluation plan to include evidence-based measurements of pre- and post-program changes in students’ knowledge, attitudes and skills towards becoming community-responsive physicians. We will measure satisfaction on an ongoing basis. We plan to offer students more opportunities for collaboration with family medicine residents, the community and other inter-professional medical learners.
